# Heat-Induced Gelation of Chickpea and Faba Bean Flour Ingredients

**DOI:** 10.3390/gels10050309

**Published:** 2024-05-01

**Authors:** Anna Mengozzi, Emma Chiavaro, Davide Barbanti, Francesca Bot

**Affiliations:** Department of Food and Drug, University of Parma, Parco Area delle Scienze 27/A, 43124 Parma, Italy; anna.mengozzi@unipr.it (A.M.); emma.chiavaro@unipr.it (E.C.); davide.barbanti@unipr.it (D.B.)

**Keywords:** legumes, faba bean, chickpeas, starch-protein interaction, food structure, rheological properties

## Abstract

This study aimed to investigate the gelling behavior of faba bean (FB) and chickpea (CP) flour between 10 and 20% (*w/w*) concentration at pH 3.0, 5.0, and 7.0. Both sources formed at pH 3.0 and 5.0 self-standing gels with 12% (*w/w*) of flour, while 16% (*w/w*) of flour was required to obtain a gel at pH 7.0. During gelling between 40 and 70 °C, a sharp increase of the elastic modulus G′ was observed in both flours, mainly due to water absorption and swelling of the starch, one of the major constituents in the ingredients. Increasing the temperature at 95 °C, G′ increased due to the denaturation of globulins and therefore the exposure of their internal part, which allowed more hydrophobic interactions and the formation of the gel. After cooling, both FB and CP gels displayed a solid-like behavior (tan δ ranging between 0.11 and 0.18) with G′ values at pH 3.0 and 5.0 significantly (*p* < 0.05) higher than those at pH 7.0, due to the lower electrostatic repulsions at pHs far from the isoelectric point. The rheological properties were supported by the water binding capacity values, confirming the better gels’ strength described by rheological analysis. These results will enhance our understanding of the role of legume flours in formulating innovative and sustainable food products as alternatives to animal ones.

## 1. Introduction

The demand for plant-based food products is constantly rising owing to the rapid growth of the global population and deteriorating climatic conditions [1] along with the consumer’s demand for sustainable and affordable ingredients. Therefore, new initiatives are required to increase the production of high-quality, functional, and sustainable plant-based ingredients [2,3,4].

Among the plant-based ingredients, pulses are affordable, nutrient-dense, and an excellent source of protein, dietary fiber, and minor nutrients such as iron and vitamins. Within the pulses family, the most relevant sources are soybeans, lentils, red kidney beans, chickpeas, and faba beans [5,6]. More specifically, faba beans can be grown in most climate areas of Europe, including Sweden, where they are extensively produced but mainly used for animal feed [7]. Faba beans are rich in starch (40–44%), fibers (11–17%), and proteins (29–36%), with the main globular fractions belonging to the proteins legumin (11S) and vicilin (7S) [8,9,10,11]. Chickpeas represent the second most cultivated legume worldwide, and India is by far the largest producer (FAO, 2016). In chickpeas, starch content is around 52–71%, fibers range between 6 and 15% [12], while lipids account for 6.5% [13]. The protein fraction ranges between 15 and 30%, and the main constituents are globulins (53–60%), glutelins (19–25%), albumins (8–12%), and prolamins (3–7%) [12].

Both faba bean and chickpea ingredients have interesting functional properties, including emulsifying, foaming, and gelling properties [10,11,12,13,14], with the latter being extremely important in food products like yogurt, cheese, puddings, and sausages. To formulate food products with desired gelling characteristics, the understanding of the gelling mechanisms as well as the molecular interactions among macro and micro constituents (i.e., starch, proteins, minerals) is of primary importance [15]. In particular, when forming a protein–starch gel during heat treatment, the main steps include starch gelatinization, which causes the swelling of the starch granules, followed by amylose leaching and loss of the granular integrity [16], together with conformational change or denaturation of the protein molecules followed by the aggregation and the consequent network formation [17]. More specifically, in legumes globulins in their native state are characterized by rigid secondary and ternary structures, whereas during heating, these polypeptide chains lose their rigid tertiary structure at the expense of improved mobility and flexibility, thus facilitating protein–protein interactions [18,19]. These phenomena can be explained by the behavior of the buried cysteine residues that are not accessible in the native conformation, and become exposed and available, leading to intermolecular cross-links upon heating [20]. 

Another factor influencing the physical structure of gels is the pH, which, by affecting the charge distribution and conformation of proteins, induces changes in the gel strength and the nature of interactions involved in the gel formation (e.g., hydrophobic interactions, hydrogen bonds, and disulfide bridges) [21,22,23].

Heat-induced gelatinization has been widely investigated in mixed protein–starch systems, containing concentrates or isolates as ingredients and starches from several pulses including lentils, chickpeas, faba and mung beans, and peas [7,10,24,25,26]. In those systems, the results have shown that during heating, protein unfolds and aggregates to form a structured matrix [22], while starch granules swell first upon hydration and leak amylose upon heating, then amylose forms a network between the starch granules [16].

Despite many studies that have been conducted on the gelation properties of different protein isolate ingredients [7,17,25,26], there is a notable absence of systematic investigations of the heat-induced gelation properties of less refined ingredients in a wide pH range. Therefore, the aim of this research was to investigate the physical properties of heat-induced gels of faba bean- and chickpea-derived ingredients at pH 3.0, 5.0, and 7.0 and in a wide concentration range (i.e., between 10 and 20% (*w/w*) flour content). In the present study, after investigating the thermal properties and the sol-gel transition of faba bean and chickpea flours at pH 3.0, 5.0, and 7.0, the rheological properties at the flour concentration able to form a gel and below and above this point were investigated.

The obtained results will provide valuable insights into their potential application in food formulations and contribute to unlocking the development of legume-based products with enhanced and tailored functional properties.

## 2. Results and Discussion

### 2.1. Thermal Properties of FB and CP Flours

**Figure 1:**
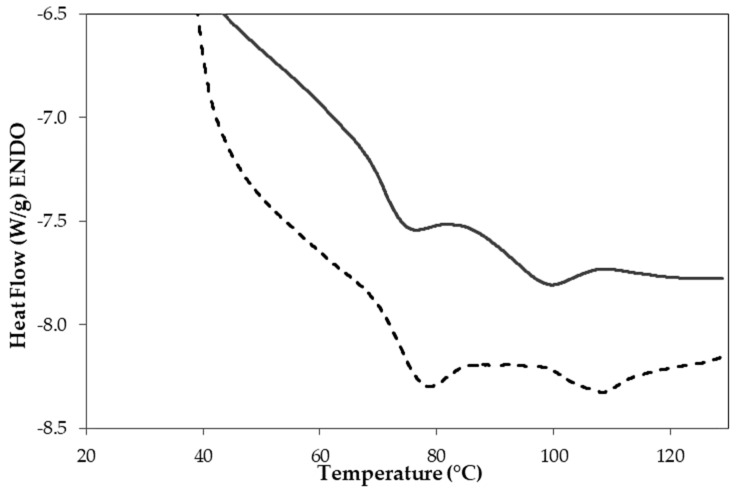
Differential scanning calorimetry thermogram of hydrated faba bean (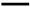) and chickpeas (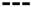) flours.

Differential scanning calorimetry analysis was performed to develop an understanding of the gelling behavior of the selected flours. In both FB and CP, two main endothermic peaks were observed in the thermogram (Figure 1). In particular, the FB sample had the first peak with T_on_ and T_off_ of 59.56 and 80.74 °C, respectively, and a T_peak_ of 74.12 °C, while the second peak had T_on_ and T_off_ of 84.72 and 104.86 °C, respectively, with a T_peak_ of 97.66 °C. A similar thermogram can be observed in CP, with the first peak having a T_on_ and T_off_ of 60.22 and 83.99 °C, respectively, and a T_peak_ of 77.32 °C. The second peak, instead, had a T_on_ and T_off_ of 95.93 and 119.36 °C, respectively, with a T_peak_ of 107.66 °C. The first endothermic peak is generally associated with starch gelatinization [27] and protein denaturation, more specifically the denaturation of (7S) vicilin [28,29]. On the other hand, the second endothermic peak can be associated with the denaturation of the (11S) legumin fraction [30]. Similar endothermic peaks have been also observed in both faba beans and chickpea ingredients [29,30,31] and the slight variation in the thermal properties of the ingredients has generally been linked to different legume varieties together with different processing parameters applied during the ingredients’ production [31,32,33]. It can be also observed that CP had a higher T_peak_ than FB, especially in the second endothermic peak. This behavior can be related to the need for a higher temperature to unfold the secondary and tertiary protein structures contained in the protein–starch network [30].

In addition, from the thermogram, it is possible to determine the enthalpy alteration linked with the gelatinization or denaturation process, which reflects the quantity of heat necessary for the thermal transition of the macro molecules under consideration and depends on the proportions of polar and non-polar interactions within the resulting aggregate [34]. In FB, the enthalpies associated with the first and the second peaks were 0.91 and 0.78 J/g, respectively, while higher values were observed in CP flours (i.e., 1.52 and 0.96 J/g, respectively, for the first and the second peaks). This indicates a significantly (*p* < 0.05) higher amount of unfolded native proteins in CP than in FB [30].

### 2.2. Sol-Gel Phase Diagram Determination of CP and FB Flours

**Figure 2:**
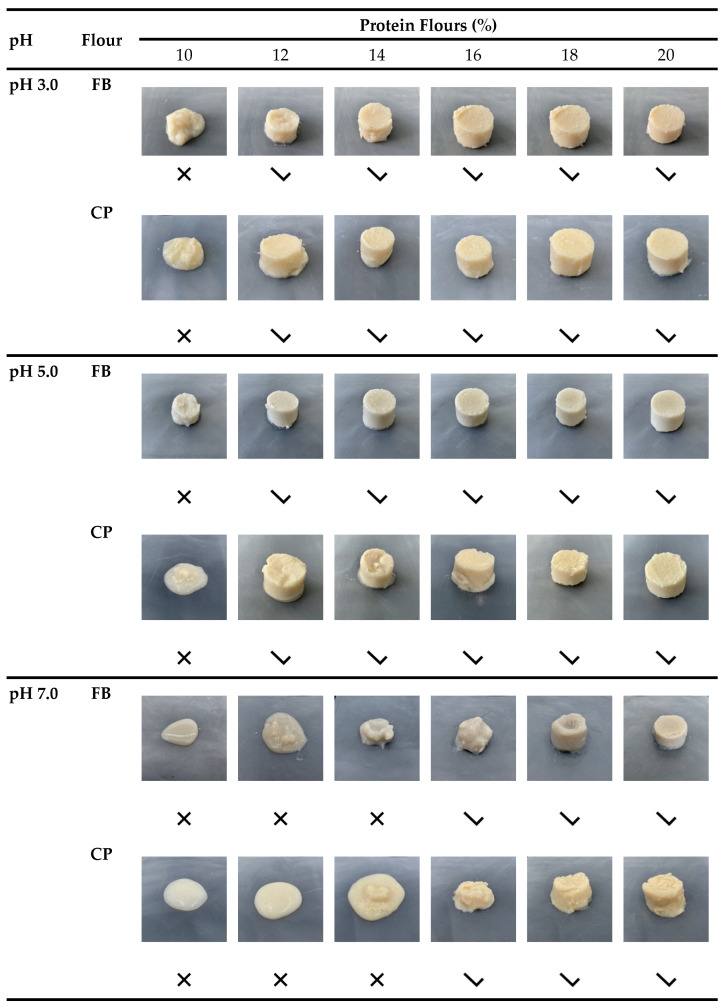
Sol-gel transition diagram of faba bean (FB) and chickpeas (CP) flours at 10, 12, 14, 16, 18, 20% (*w/w*) and pH 3.0, 5.0, and 7.0 Symbol 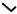 indicates a gel-like structure and symbol 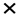 indicates a liquid or aggregate-like structure.

Protein–starch suspensions when heat treated generally undergo phenomena like phase separation, aggregation, and gelation, and these modifications are strongly affected by many factors including protein type, concentration, and pH [23,24,25,26,27,28,29,30,31,32,33,34,35]. Figure 2 shows the sol-gel phase diagram of FB and CP at increasing flour concentrations and pH 3.0, 5.0, and 7.0. At pH 3.0, the minimum gelling concentration (MGC), defined as the concentration at which the gel is self-standing and does not flow when the tubes are inverted, was 12% (*w/w*) for both FB and CP. The gels appeared with a smooth and homogeneous texture and, when increasing the flour concentration up to 20% (*w/w*), both FB and CP displayed a self-standing and compact structure. At pH 5.0, the MGC values were similar to the ones observed at pH 3.0, with a value of 12% (*w/w*) in both FB and CP, while at pH 7.0, the MGC increased to 16% (*w/w*) in both the flours. The higher MGC values at pH 7.0 compared to pH 3.0 and 5.0 could be attributable to multiple factors, including the pH, which was set at values far from the isoelectric point (IP) (i.e., pH 4.3 and 5.0 for FB and CP, [23,36,37,38], the strong electrostatic repulsions among the proteins, and the presence of starch, which has been gelatinized during the heat treatment [7,39,40,41]. Similar MGC values have been also found for pea, faba bean, and soybean protein isolates, which at neutral pH had an MGC of 18%, 14%, and 16%, respectively [26,37,42,43].

### 2.3. Water Holding Capacity of Gelled FB and CP Flours

**Figure 3:**
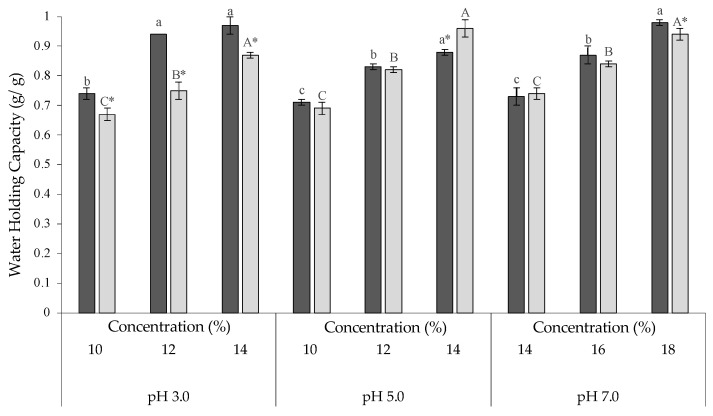
Water holding capacity of faba beans (FB) (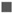) and chickpeas (CP) (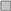) gels at 10, 12, and 14% at pH 3.0 and 5.0, and 14, 16, and 18% at pH 7.0. Different lowercase (a, b, c) (FB) and uppercase (A, B, C) (CP) letters indicate significant (*p* < 0.05) differences among the same source and same pH. Differences between sources (FB and CP) within the same pH and concentration are indicated with *.

Water holding capacity (WHC) indicates the ability of an ingredient to interact with water under conditions where water is limited [44] and it is generally affected by both intrinsic (e.g., protein structure, conformation, amino acid composition, hydrophobicity, hydrophilicity, and starch content) and extrinsic (e.g., pH, temperature, and ionic strength) factors [45,46]. Generally, low WHC is often associated with a weak gel structure not able to hold water [47] and in starch-based gels, the presence of proteins can affect the WHC and the water distribution within the matrix [45,46,47,48]. The WHC of CP and FB have been investigated at the MGC, the concentration below the MGC (−2% MGC), and the concentration above the MGC (+2% MGC) in all the pH range considered, in order to gain a better understanding of ability of the selected ingredients to hold water (Figure 3). At pH 3.0, the WHC of FB was significantly (*p* < 0.05) higher than CP with values ranging between 0.74 and 0.97 g/g for FB gels, and between 0.67 to 0.87 for CP gels. Similar results have been also observed in legume flours such as chickpeas and lentils, which had a water binding capacity ranging between 0.72 and 1.0 g/g at neutral pH [14].

Moving to pH 5.0 and 7.0, the WHC had slightly lower WHC values compared to those found at lower pH, with values ranging between 0.67 to 0.98 g/g in both FB and CP. The higher WHC at pH 3.0 than at pH 5.0 can be attributed to proteins and their solubility [49]. Indeed, at pH close to the isoelectric point (i.e., pH 4.3 and 5.0 for FB and CP, respectively) proteins aggregate, thus allowing an increase in water binding capacity [50,51].

### 2.4. Rheological Properties of FB and CP Gels

**Figure 4:**
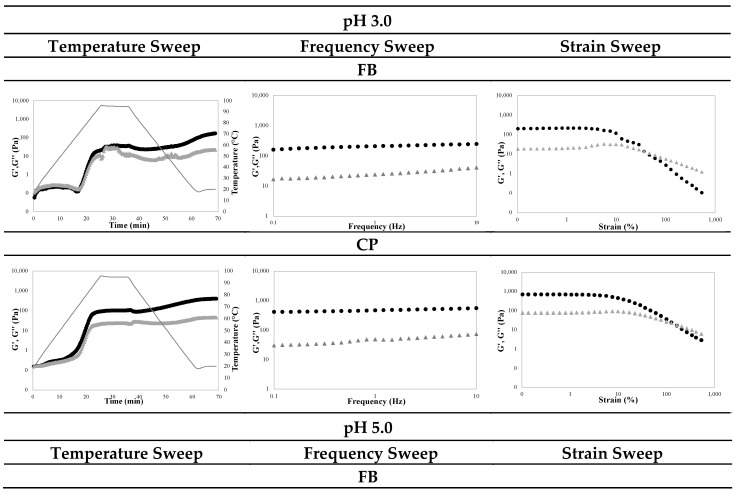

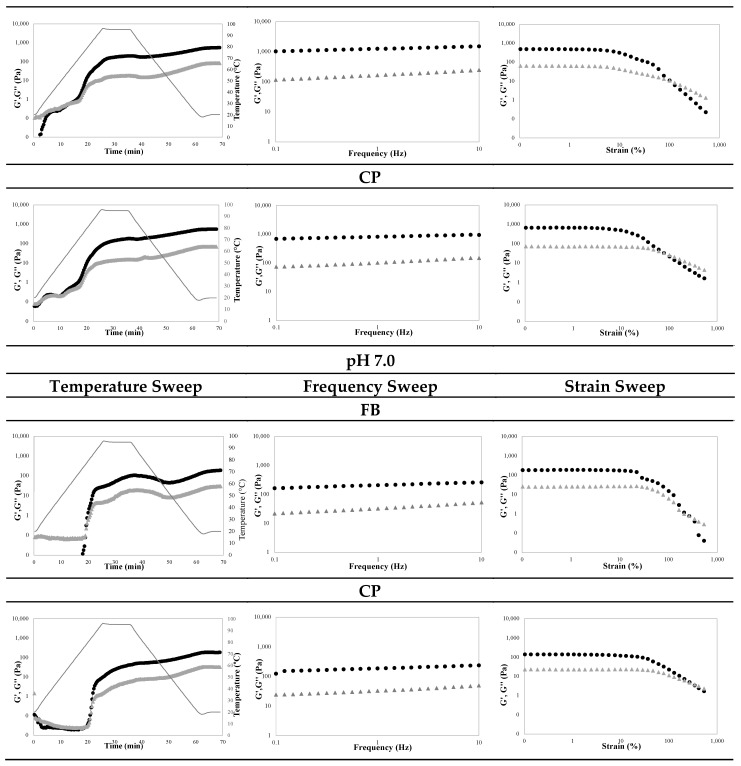
Temperature, frequency, and strain amplitude sweeps of faba bean (FB) and chickpea (CP) gels at pH 3.0, 5.0, 7.0 at the minimum gelling concentration (MGC). G′: black point symbols, G″: grey triangle symbols.

The gelation behaviors of faba bean and chickpea flour suspensions were studied for the MGC, MGC−2%, and MGC+2% by measuring G′, G″, and tan δ as a function of time upon and after the heat treatment (Figure 4 and Figure S1, and Table 1).

During the temperature sweep test between 65 and 85 °C, a steep increase of the elastic modulus (G′) was observed in both FB and CP (Figure 4, left panel). The results reflect the DSC thermographs (Figure 1), where the first peak ranged between 60 and 85 °C for both FB and CP. The rapid G′ and G″ increase can be mainly attributed to starch gelatinization, which is characterized by water absorption and swelling of the starch granules. At the same time, the subsequent decrease in G′ could be due to loss of crystallinity, uncoiling, dissociation of double helices, and leaching of amylose in the continuous phase of the starch [52,53]. Increasing the temperature to 75 °C, a slight increase in G′ in both sources has been observed, while during the holding time at 95 °C, both G′ and G″ did not change. In the final cooling step, G′ further increased and reached the highest value, indicating the presence of a gelled structure. The increase of G′ can be attributed to the formation of hydrogen bonds, which stabilizes and strengthens the matrix [54,55], and to the presence of starch granules and fiber particles, which acted as active fillers and reinforced the gel [7,8,9,10,11,12,13,14,15,16,17,18,19,20,21,22,23,24,25,26,27,28,29,30,31,32,33,34,35,36,37,38,39,40,41,42,43,44,45,46,47,48,49,50,51,52,53,54,55,56]. In addition, also the proteins fraction play a role in the gel formation. Indeed, an increase of the temperature up to 95 °C caused proteins denaturation, the exposure of their interior part, and, as a consequence, the formation of hydrophobic interactions and the formation of a network typical of a gel structure [57,58]. Similar temperature sweep profiles have been observed between the two sources at all the pH considered (Figure 4).

Interestingly, after cooling, different G′ values were found between FB and CP: at pH 3.0, FB displayed always lower G′ than CP. More specifically, at 12% (*w/w*) flour content, corresponding to the MGC, G′ for FB and CP was 159 and 869 Pa, respectively (Table 1). At pH 5.0 and below the MGC (i.e., 10% (*w/w*) flour content), FB had a significantly (*p* < 0.05) lower G′ than CP, while, when increasing the flour content, only slight differences can be observed among the two sources. Moving to pH 7.0, G′ values in FB and CP were lower than the ones observed at pH 5.0 and 3.0, indicating that at pHs far from their IP have a minor contribution to the gels’ strength, which is mainly attributed to the gelatinized starch [57]. This behavior is confirmed by no significant (*p* > 0.05) differences in G′ between FB and CP at pH 7.0 and flour concentrations of 14% and 16%. At pH 7.0 and 14% (*w/w*) flour concentration, G′ ranged between 111 and 113 Pa in FB and CP gels. When increasing the flour concentration up to 16% (*w/w*), which corresponds to the MGC+2 point, G′ increased in both FB and CP gel and reached 194 and 413 Pa, respectively. In addition, the results highlighted that the rheological properties are correlated with the WHC (Figure 2), as already shown by other authors in various mixed systems [15,59,60].

The tan δ values of FB and CP gels in all the pH ranges were below 0.25, with values ranging between 0.11 and 0.18 (Table 1). This indicates that the gels had a solid-like behavior typical of weak gels upon heating, which can be attributed to the chemical composition (with starch and proteins as the main macronutrients) and pH. Similar tan δ values have been also observed in gelled systems formulated with different legume flours or protein isolate fractions from peas [57,58].

After the heat-induced gelation step, the G′ and G″ dependencies on frequency were determined by a frequency sweep test in order to have a better understanding of the type of gel formed (Figure 4). In both FB and CP gels at all the selected pHs, G′ was higher than G″ over the full frequency range, thus indicating the predominance of the elastic component. In addition, both moduli were constant over this frequency range and did not display any dependency on frequency. It can be also observed that, at high frequency, the nearly flat line could indicate the presence of a broad relaxation spectrum that is typical for a disordered system [61]. Several studies have observed similar behavior in gels formulated with plant proteins (e.g., pea) or starch-based batter added with proteins [7,25,49,62]. However, there is no full agreement on the results available in the literature for gels formulated with legume flours or starch–protein-based ingredients. Some authors have reported that the addition of protein in starch-based systems decreased the G′ and G″ values [63,64,65,66,67], whereas others have observed an increase of G′ and G″ values only in a certain frequency range [68,69,70,71]. Therefore, further studies are still needed in this area to understand the structural properties of the protein–starch gel formed in function on the frequency applied.

Moving to the strain sweep test in the right panels of Figure 4, it is possible to observe that in all the pH ranges investigated and within the linear viscoelasticity region (LVE), both FB and CP had a G′ always higher than G″, thus indicating a solid-like behavior of the ingredients [72]. By increasing the strain, both the G′ and G″ dropped steeply, thus indicating that the gels become more fluid-like, due to the disruption of the initial structured network [72]. In both FB and CP, the critical strain range was lower than 10% and similar values have been found in yellow pea ingredients with different protein (19–87%) and carbohydrates (60–3%) content [57] or in starch-based systems [73,74,75,76].

## 3. Conclusions

The results of this study provide insight into the gelling properties of faba bean (FB) and chickpea (CP) flours at pH 3.0, 5.0, and 7.0. The thermal analysis revealed a distinct pattern in the starch gelatinization and protein denaturation temperatures among the different flours, which can be attributed to different macronutrient compositions together with starch granule characteristics and different protein fractions. The MGC values for both FB and CP were identified at 12% (*w/w*) flour content at pH 3.0 and 5.0, while the value increased up to 16% (*w/w*) at pH 7.0, due to the low ability of FB and CP proteins to form a gel at pH far from the isoelectric points.

The rheological properties of the heat-induced gels showed higher G′ at pH values close to or below the isoelectric point (i.e., pH 3.0 and 5.0) than pH 7.0 with a tan δ, indicating a weak gel structure. The greater ability to enhance gel structure at lower pH (3.0, and 5.0) can be associated with proteins being closer to their isoelectric points and their ability to associate to each other through van der Waals, hydrophobic, or hydrogen bonding as well as with the ability of starch in reinforcing the matrix. Slight differences can be observed between the sources, with a slight tendency of FB to form gels with higher elastic moduli.

These new insights on how pH and flour concentration can influence the rheological and structural properties of gels made of different flour sources will support the further development of innovative food formulations spanning from fermented to neutral products.

## 4. Materials and Methods

### 4.1. Materials

Faba bean (FB) flour, with the following composition of carbohydrates 52%, fibers 21%, proteins 30%, fat 1.9%, and minerals 0.03% was provided by Ingredion (Hamburg, Germany). Chickpea (CP) flour was provided by Muller’s Muhle GmbH (Gelsenkirchen, Germany), with the following macronutrient content, carbohydrate 49%, fiber 11%, proteins 21% and fat 6.5% and minerals <0.1%. All the reagents used in this study were of analytical grade and supplied by Sigma–Aldrich (St Louis, MO, USA) unless otherwise stated.

### 4.2. Differential Scanning Calorimetry (DSC)

Thermograms of the FB and CP flours were obtained using a differential scanning calorimeter (DSC) (DSC Q100, TA Instruments, New Castle, DE, USA) following the method of [29]. Indium and mercury were used to calibrate the instrument and an empty pan was used as reference. All the flours were weighed (5.2–8.6 mg) into hermetic aluminium pans and ~ 10 mg of distilled water was added to hydrate the flours for 24 h at 4 °C. Flour suspensions were equilibrated at 20 °C and then heated from 20 to 130 °C at 5 °C/min. DSC curves were analyzed with Universal Analysis Software (Version 3.9A, TA Instruments) to obtain the enthalpy change for transition (ΔH, J/g), the onset temperature of transition (T_on_, °C), offset temperature of transition (T_off_, °C), and peak temperature at the maximum (T_peak_, °C) for the main events during heating transitions. 

### 4.3. Minimum Gelling Concentration (MGC)

Aqueous solutions were prepared by adding 10, 12, 14, 16, 18, and 20% (*w/w*) of flour (FB and CP) and deionized water. The suspensions were magnetically stirred for 3 h at room temperature and after 2 h and 30 min of stirring, pH was adjusted to 3.0, 5.0, and 7.0 by adding 1 M HCl or 1 M NaOH. To induce gelation, FB and CP suspensions were transferred in 50 mL sealed falcon tubes and subjected to heat treatment in a water bath (95 °C for 1 h), followed by a first cooling in a water bath (15 °C for 10 min). The heat-treated samples were then stored at 4 °C for 24 h before analysis. The minimum gelling concentration (MGC) was determined as the sample with the lowest concentration that did not flow when the tubes were inverted [73].

### 4.4. Gel Analyses

The samples considered in the following analyses were the ones identified as the MGC, the concentration below the MGC (−2% MGC), and the concentration above the MGC (+2% MGC).

#### 4.4.1. Water Holding Capacity

Samples were prepared with the same method as described in Section 2.3. The water binding capacity (WHC) was determined after gelation induced by heat treatment for all the pH considered (pH 3.0, 5.0, and 7.0). The samples were left to stand overnight at 4 °C and were weighed (W_i_), before centrifugation at 3000× *g* at 4 °C for 10 min (Centrifuge 5810 R, Eppendorf, Fisher Scientific, Waltham, MA, USA). The remaining pellets were then weighed (W_r_). The samples were measured in duplicate, and WHC was calculated using the following equation [25]:

(1) $$WHC=\frac{W_{r}}{W_{i}}$$

#### 4.4.2. Rheological Measurements

Rheological properties were performed in an MCR102 rheometer (Anton Paar, Graz, Austria) equipped with a CC-27 concentric cylinder geometry. Samples were prepared with the same method as described in Section 4.3. Gelling occurred within the cylinder and to prevent solvent from evaporating during heating, a solvent trap was placed on top of the probe. The samples were sequentially exposed to temperature, frequency, and amplitude sweep. For temperature sweeps, the samples were heated from 20 to 95 °C at a rate of 3 °C/min. Then, the samples were kept at 95 °C for 10 min before cooling back to 20 °C. The temperature was kept at 20 °C for 5 min. The storage (G′) and loss (G”) moduli were recorded as a function of temperature at a frequency of 1 Hz and a strain amplitude of 1%. Subsequently, the gels were subjected to a frequency sweep at 1% deformation with a frequency increase from 0.01 to 10 Hz. Amplitude sweeps were carried out at a frequency of 1 Hz and amplitude strain from 0.1% to 1000% to determine the linear viscoelastic region. G′ and G″ dependency on temperature, frequency, and amplitude were recorded. The three tests followed the method of [74]. Data on rheological measurements were run at least in triplicate.

#### 4.4.3. Statistical Analyses

The results are the average of at least three measurements carried out on at least three replicated experiments from the same sample batch. Data are reported as mean value ± standard error. Statistical analysis was performed using the SPSS (version 28.0.1.0 SPSS Inc., Chicago, IL, USA) statistical software package. Levene’s test to check the homogeneity of variance, a one-way analysis of variance (ANOVA) and Tukey’s postdoc significant difference test (HSD) at a 95% confidence level (*p* < 0.05) to identify statistically significant differences among means. Dunnett’s T3 test was used when the variances were not equal.

## Figures and Tables

**Table 1:** Average elastic moduli (G′) and loss factors (tan δ) of the gelled faba bean (FB) and chickpea (CP) gels at 10, 12, and 14% pH 3.0 and 5.0, and 14, 16, and 18% at pH 7.0. The elastic moduli and loss factor correspond with the values of the last data point from the temperature sweeps and the critical strains. Data are taken from the temperature sweeps. Different lowercase (^a^, ^b^, (FB) and uppercase (^A^, ^B^, ^C^) (CP) letters indicate significant (*p* < 0.05) differences among the same source and same pH. Differences between sources (FB and CP) within the same pH and concentration are indicated with *.

		**G′ (Pa)**	**tan δ (-)**	**G′ (Pa)**	**tan δ (-)**	**G′ (Pa)**	**tan δ (-)**
**Concentration (%)**		**10**		**12**		**14**	
**pH 3.0**	FB	32.21 ± 12.66 ^b^*	0.15 ± 0.05 ^a^	159.01 ± 38.57 ^b^*	0.14 ± 0.04 ^a^	1505.39 ± 117.52 ^a^	0.15 ± 0.06 ^a^
	CP	631.42 ± 23.83 ^C^	0.11 ± 0.01 ^A^	869.75 ± 35.31 ^B^	0.11 ± 0.00 ^A^	1254.51 ± 100.47 ^A^	0.11 ± 0.00 ^A^
**Concentration (%)**		**10**		**12**		**14**	
**pH 5.0**	FB	424.95 ± 48.20 ^b^	0.14 ± 0.00 ^a^*	552.65 ± 131.54 ^b^	0.15 ± 0.00 ^a^	1144.25 ± 83.8 ^a^	0.14 ± 0.01 ^a^
	CP	238.07 ± 29.14 ^C^*	0.16 ± 0.01 ^A^	590.06 ± 93.26 ^B^	0.13 ± 0.01 ^B^*	1038.40 ± 66.88 ^A^	0.13 ± 0.01 ^B^*
**Concentration (%)**		**14**		**16**		**18**	
**pH 7.0**	FB	113.75 ± 29.02 ^a^	0.14 ± 0.05 ^a^	186.91 ± 44.39 ^a^	0.15 ± 0.00 ^a^	194.03 ± 29.31 ^a^*	0.16 ± 0.01 ^a^
	CP	111.02 ± 14.55 ^C^	0.18 ± 0.01 ^A^	162.82 ± 21.31 ^B^	0.18 ± 0.01 ^A^	413.85 ± 58.0 ^A^	0.18 ± 0.01 ^A^

## Data Availability

The data that support the findings of this study are available from the corresponding author upon reasonable request.

## Supplementary Materials

The following supporting information can be downloaded at: https://www.mdpi.com/article/10.3390/gels10050309/s1, Figure S1: Temperature, frequency, and strain amplitude sweeps of faba bean (FB) and chickpea (CP) gels at 10 and 14% for pH 3.0 and 5.0, and 14, and 18% at pH 7.0. (excluding the MGC). G′: black point symbols, G″: grey triangle symbols.
