# Supplementary material for: Heat-Induced Gelation of Chickpea and Faba Bean Flour Ingredients
Source: Gels. 2024 May 1;10(5):309. doi: 10.3390/gels10050309 (PMC11121298; doi:10.3390/gels10050309)
Supplement: Supplementary file 1 [file gels-10-00309-s001.zip › gels-2964468-supplementary.pdf]

pH 3.0

Temperature Sweep	Frequency Sweep	Strain Sweep

10%

FB

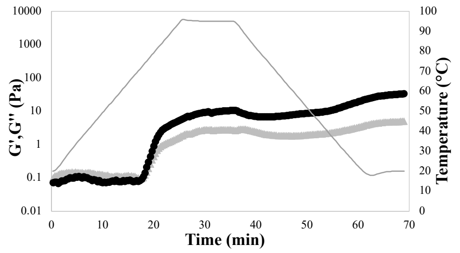

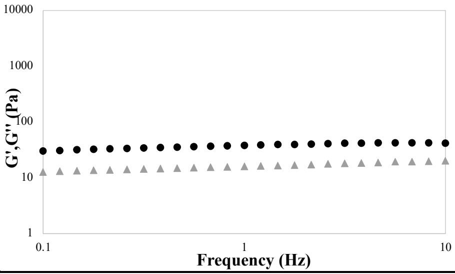

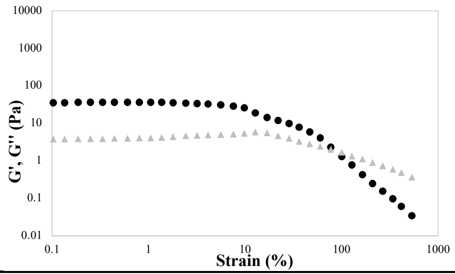

CP

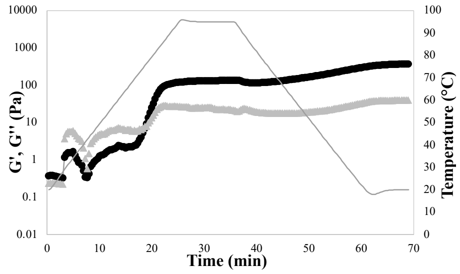

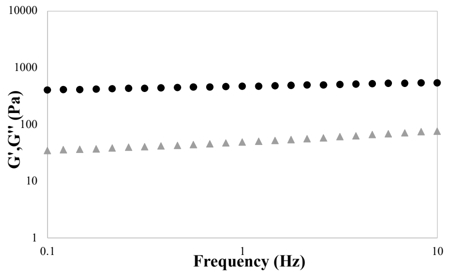

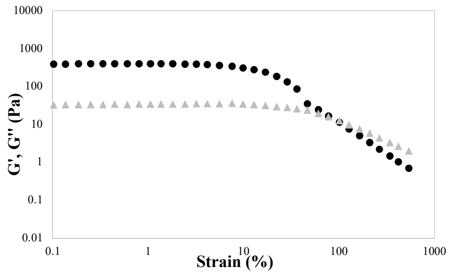

14%

FB

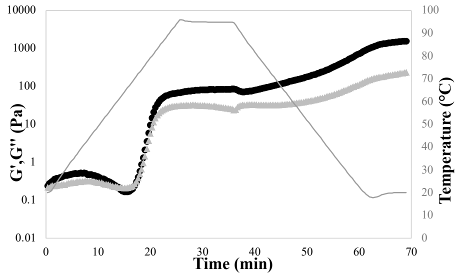

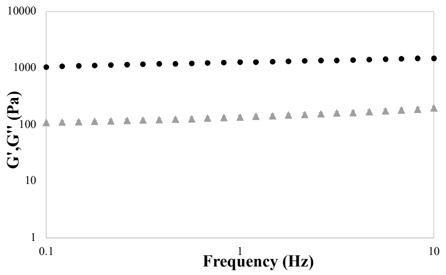

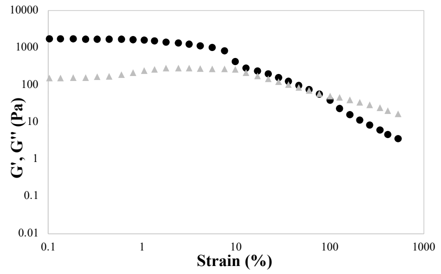

CP

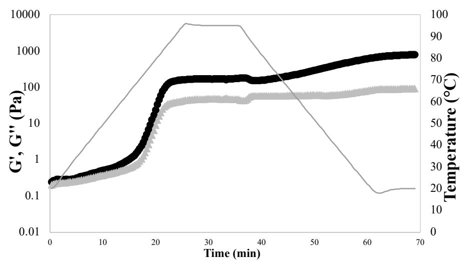

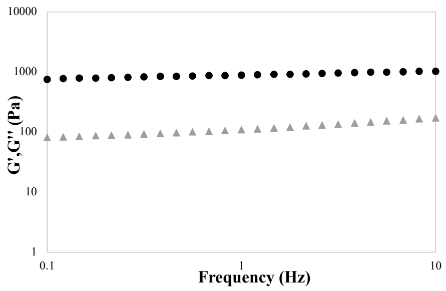

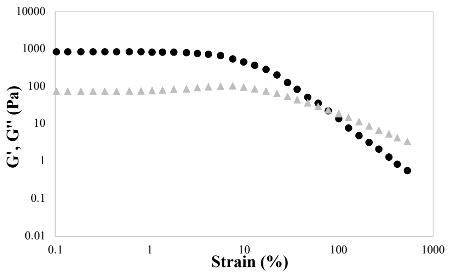

pH 5.0

Temperature Sweep	Frequency Sweep	Strain Sweep

10%

FB

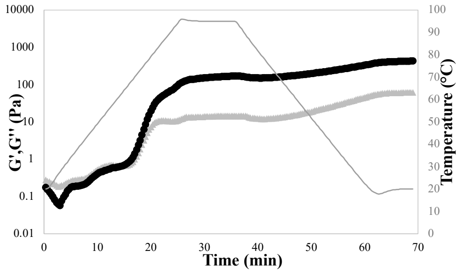

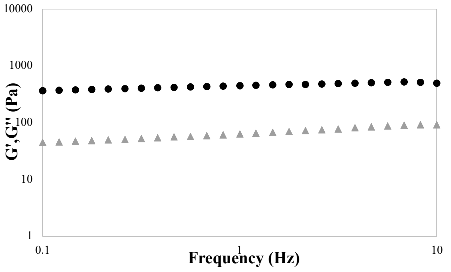

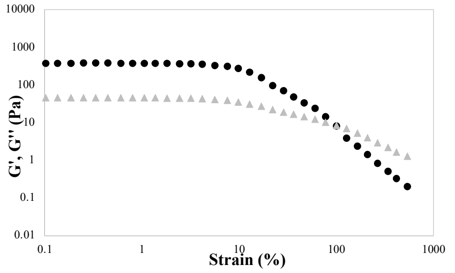

CP

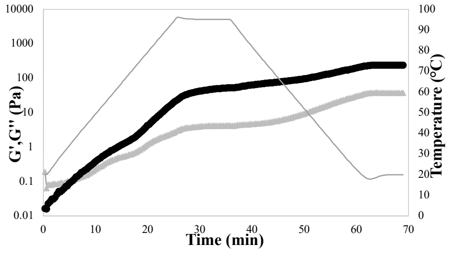

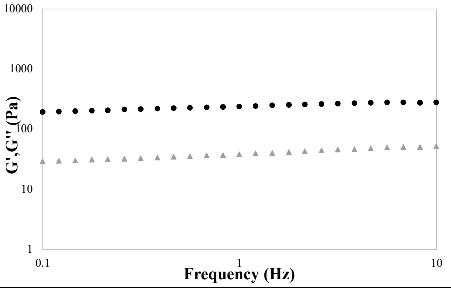

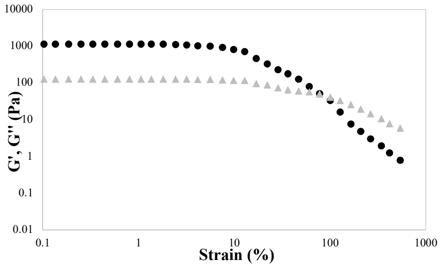

14%

FB

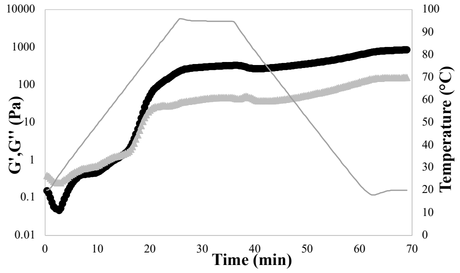

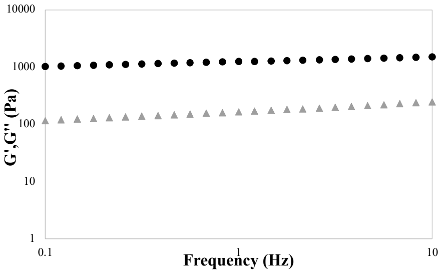

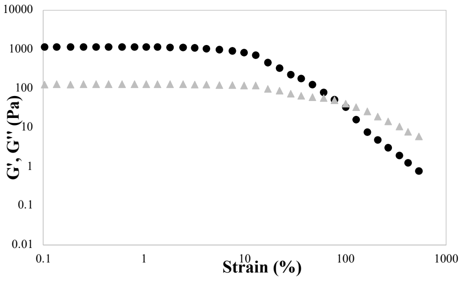

CP

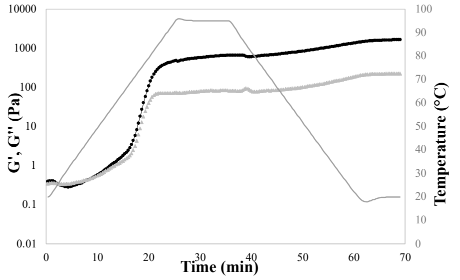

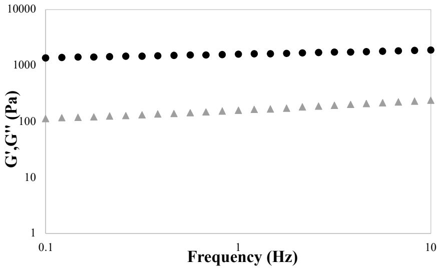

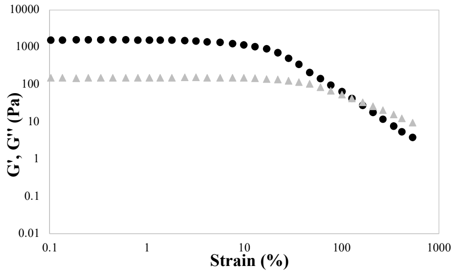

pH 7.0

Temperature Sweep	Frequency Sweep	Strain Sweep

14%

FB

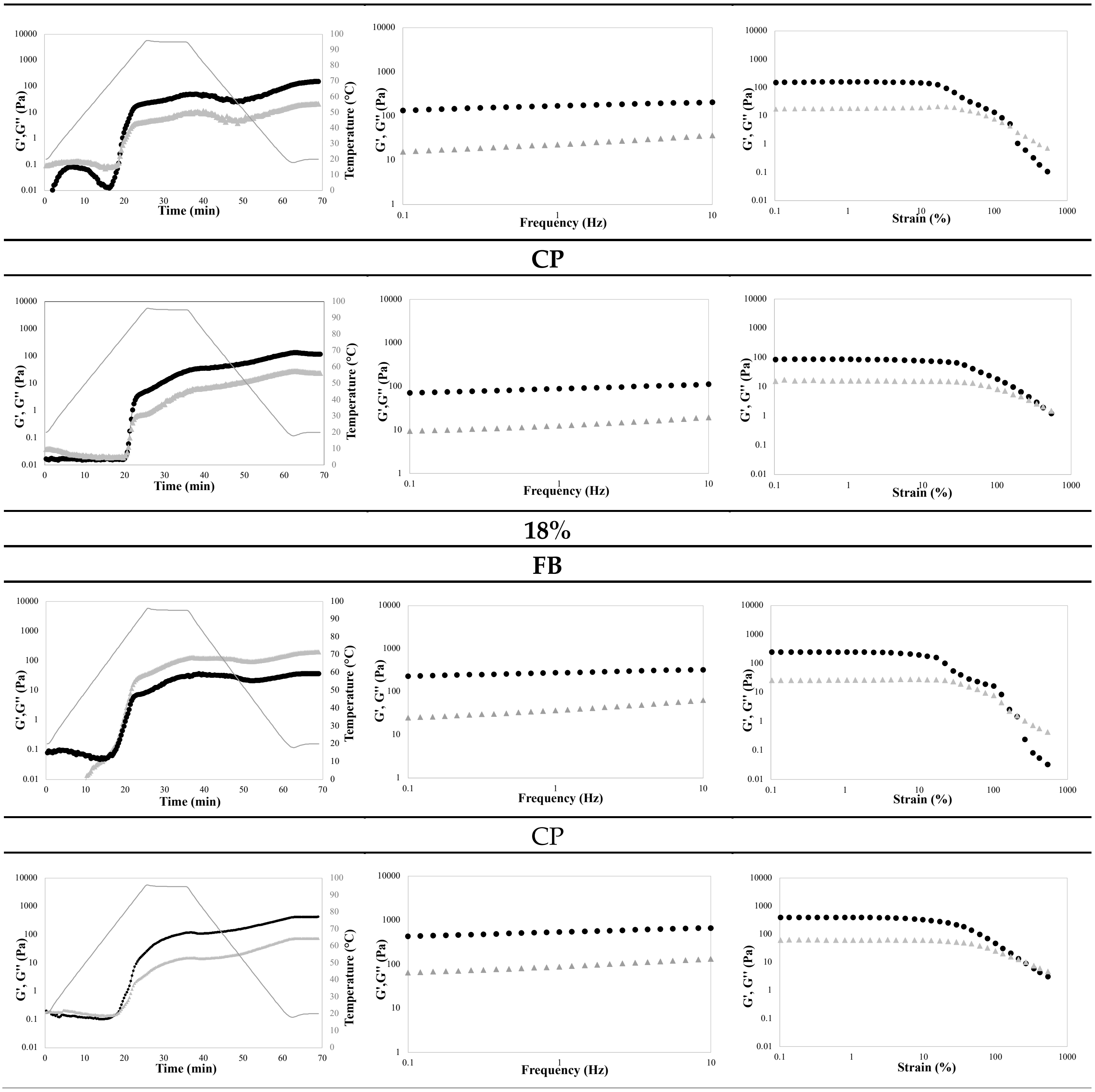

Figure S1. Temperature, frequency, and strain amplitude sweeps of faba bean (FB) and chickpea (CP) gels at 10 and 14% for pH 3.0 and 5.0, and 14, and 18% at pH 7.0. (excluding the MGC). G′: black point symbols, G′′: grey triangle symbols.
